# Wood Biomass Ash (WBA) Used in Conjunction with Post-Fermentation Mass (PFM) as a Way to Stabilize Soil Properties

**DOI:** 10.3390/ma18225176

**Published:** 2025-11-14

**Authors:** Elżbieta Rolka, Mirosław Wyszkowski, Andrzej Cezary Żołnowski, Anna Skorwider-Namiotko, Radosław Szostek

**Affiliations:** Department of Agricultural and Environmental Chemistry, Faculty of Agriculture and Forestry, University of Warmia and Mazury in Olsztyn, Łódzki 4 Sq., 10-727 Olsztyn, Poland; miroslaw.wyszkowski@uwm.edu.pl (M.W.); andrzej.zolnowski@uwm.edu.pl (A.C.Ż.); anna.namiotko@uwm.edu.pl (A.S.-N.); radoslaw.szostek@uwm.edu.pl (R.S.)

**Keywords:** waste, WBA (wood biomass ash), PFM (post-fermentation mass), soil pH, soil sorption complex, macronutrients, soil salinity

## Abstract

Nowadays, waste that can be used for environmental purposes, such as WBA (woody biomass ash), is particularly important. The presented research assessed the effect of soil application of WBA in conjunction with PFM (post-fermentation mass) on the stabilization of soil properties. WBA was applied in three increasing doses (0.5, 1.0, and 1.5 HAC). PFM was applied as follows: ULF (unseparated liquid fraction), SSF (separated solid fraction), and SLF (separated liquid fraction). PFM doses were balanced with the amount of nitrogen introduced into the soil. The study was based on a pot experiment with maize. The applied doses of WBA had a highly significant and positive effect on the stabilization of basic soil properties. After WBA application, hydrolytic acidity decreased (by 30%), soil pH increased (by 1.83 units), total base cation increased (by 66%), available potassium (by 119%), phosphorus (by 44%), and magnesium content (by 38%) as well as electrolytic conductivity increased (by 11%). Furthermore, an increase in soil carbon content and an improvement in the carbon-to-nitrogen ratio were noted. These observed results were further enhanced by the simultaneous application of WBA and the used PFM fractions, of which the liquid fractions (ULF and SLF) had the strongest effect.

## 1. Introduction

The current development of civilization imposes new challenges on societies related to the rational use of resources and the rational management of generated waste. The increasingly depleted global resources are forcing a trend towards recovering raw materials from waste products. Such products currently include WBA and PFM. In many countries, including Poland, WBA is still treated as waste [1] and disposed of in landfills, which has become increasingly expensive in recent years [2]. It is estimated that 476 million tons of WBA could be obtained from biomass combustion in a single year [3]. According to Ike et al., 2025 [4], the amount of WBA obtained could be over 1.5 times greater than that calculated based on fuel properties. Depending on the raw materials used in the fermentation processes, PFM can be an organic fertilizer or, at best, a problematic by-product. In the case of fermentation of typical agricultural waste (corn silage, liquid manure, etc.), PFM is called an organic fertilizer [5,6]. However, when biodegradable municipal waste, not included in the list of safe feedstocks [7], is fermented, PFM is referred to as waste. This waste is quite problematic to manage due to its high water content and often high salinity. In recent years, we have observed intensive development of the biogas industry in Europe. In many countries, biogas production has been promoted as a means of reducing greenhouse gases [8]. The implementation of the European Green Deal aims to accelerate the expansion of biogas plants and encourage the development of this type of investment [9]. Due to the growing interest in green energy from renewable sources, the amount of both WBA and PFM is expected to increase [9]. This makes it all the more important to manage them rationally, so that they can transform from burdensome waste into valuable materials. Both products have already attracted the attention of scientists, who attribute great potential to both WBA and PFM in environmental applications [9,10,11]. The composition of WBA is highly variable [3,12], depends on the type of burnt wood [4,13,14], origin, time, and storage conditions [15], and is most often dominated by K, Ca [3,16], P, and S [16]. This material is generally characterized by high alkalinity [10,17,18], rich chemical composition, except for nitrogen (N) [19,20], and a dusty form, which may be a nuisance during soil applications. Low N content may reduce the potentially positive effect of WBA on plant development [20]. Furthermore, after burning wood chips, WBA must be extinguished with water due to unburned organic matter and the possibility of spontaneous combustion. PFM, on the other hand, is most often characterized by a liquid consistency and a relatively high content of macro- and microelements [21,22], including easily assimilable plant nutrients, mainly N [9,23]. However, some studies indicate too low concentrations of P and S available to plants, which necessitates the use of additional mineral fertilizers [21]. The high water content in PFM (95.6–97.9%) may be a factor limiting its use [24]. In recent years, biogas plants have been separating PFM into solid and liquid fractions to reduce the volume for transport [25], which generates additional economic outlays. Among these fractions, the most commonly obtained are a solid mass visually similar to manure and a liquid fraction derived from drying processes. Some biogas plants, in turn, separate three fractions: ULF, SSF, and SLF. SSF and SLF are typically the fractions obtained after drying the initial PFM.

Given these general characteristics, these two materials can complement each other well. WBA can be a material rich in macro- and micronutrients, while PFM can supplement this composition with N content and moisturize WBA, thus reducing environmental risks associated with WBA dusted during soil application. Scientific studies indicate the positive impact on soil of using both WBA [4,10] and PFM separately [9,11,26]. WBA contributes to increasing soil pH [10,12,17,19,27,28], carbon content, and improving the sorption complex [10]. It improves soil physical properties, soil structure, and water retention [12]. Moreover, it increases the availability of available macroelements (P, K, Mg) [10,28], exchangeable cations (Ca_2_^+^, K^+^, Mg_2_^+^, Na^+^) [29] and also diversifies the content of S, Zn [17,30], Fe, Mn [30] and Cu [19] in the soil. The addition of WBA increases the Na content [17] and electrolytic conductivity (EC) but without excessive increase in salinity [10,29]. WBA is considered a rich source of K [4,27,28], and therefore can be a sustainable alternative to conventional potassium fertilizers [4,27]. Due to its properties and impact on soil chemical composition and nutrient supply [20], the use of WBA is particularly useful in managing the fertility of forest and acidic soils [2,20,29,30], replacing liming [12,17,19] and supplementing nutrients removed during harvesting [28]. The effects of WBA soil application are observed even up to several years after application [19]. PFM obtained from good quality materials typically improves the chemical and physical properties of soil, increasing its nutrient and organic matter content [9,11,26,31]. Regardless of the fraction, PFM generally improves soil properties and demonstrates the ability to regenerate it due to the improvement of biological properties and soil structure, as well as supplying the soil with TC and nutrients [32]. The solid fraction has a positive effect on the content of organic matter in soil [33,34] and the cation exchange capacity (CEC), and due to the higher concentration of components, it has a higher fertilization potential than the liquid fraction [34]. The liquid fraction can reduce N losses caused by ammonia emissions [23] and is additionally characterized by poor biodegradability due to the presence of humic substances [25]. The effects of PFM use are influenced not only by its fraction, but also by the input material [9,26] and soil type [9]. Well-fermented PFM, applied at appropriate doses and times, usually shows high potential in the short and medium term and gives more favorable results compared to nitrogen mineral fertilizers [35]. The fertilizing value of PFM depends, among other things, on its effect on the amount of available nutrients in the soil [36].

Much less literature data concerns the combined use of these materials or the application of various PFM fractions, particularly SSF in conjunction with WBA, and the impact of these mixtures on soil. Available studies indicate that the combined use of PFM and BA (biomass ash) in field conditions, at doses of 30 m^3^ PFM ha^−1^ and 48 q BA ha^−1^, can contribute to CO_2_ sequestration in soil and increase soil concentrations of N, P, K, and Mg [14], which is particularly important for mineral-poor soils [31]. The combined use of PFM and biochar (at doses of 5% and 1%) can alleviate soil salinity caused by the application of PFM alone [31]. Furthermore, the combined application of BA and PFM [14] and biochar and PFM [31] or WBA and PFM (at currently considered doses) [37] improves plant growth, yield, and chemical composition.

Deteriorating soil quality, the deficiency of organic matter and plant nutrients, the depletion of the sorption complex, and the observed soil acidification should encourage the natural use of materials that tend to improve these properties. However, it should be remembered that both materials under consideration (WBA and PFM), in addition to their positive impact on soil, may promote soil salinization [10,26] and excessive nutrient runoff [8], which may limit their natural use [26]. Excessive ammonia introduced from PFM may negatively affect earthworms, collembolans, and nematodes inhabiting the soil [22]. Negative changes always occur faster in light soils with low pH and poor buffering properties [9]. Due to the above, studies were undertaken in which the hypothesis was put forward that the selection of PFM fractions for combined use with WBA is important and results in a differentiated effect on the basic properties of acidic soil. The aim of the study was to assess the effect of the combined application of WBA and three separated PFM fractions (ULF, SSF and SLF) on the soil pH, sorption complex and salinity, the content of total carbon forms (TC) and nitrogen (TN), their ratio and the content of available forms of P, K and Mg in the soil.

## 2. Materials and Methods

### 2.1. Experimental Assumptions

**Figure 1:**
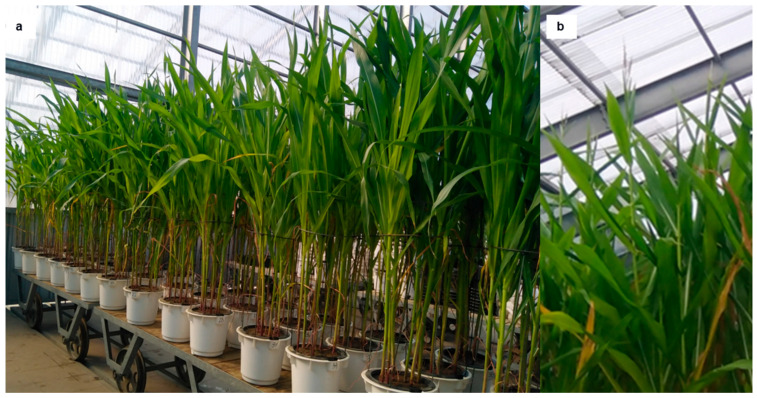
Maize plants; (**a**) 48th day of growth; (**b**) 63rd day of growth, panicles visible.

This study examined the effect of soil application of WBA and simultaneous application of PFM on selected soil properties. The study was based on a pot experiment conducted in 2024 in a vegetation hall located at the University of Warmia and Mazury in Olsztyn, Poland. The test plant was maize cultivar Garantio, grown from 10 May to 11 July (62 days), to the initial panicle stage (BBCH53) (Figure 1).

The study included four series in which WBA and three PFM fractions were used, including ULF, SSF and SLF according to the adopted scheme (Table 1). WBA was applied at three rates: 0.5, 1.0, and 1.5 HAC. WBA doses were determined based on the alkalinity of this material (26.93%) and hydrolytic acidity (26.25 mmol kg^−1^) of the soil used in the study. The alkalinity of the WBA was compared to the alkalinity of total calcium oxide. Converted to g WBA per kg of soil, these were 2.436, 4.872, and 7.308. A control treatment (without WBA) was also considered in each series. The doses of individual PFM fractions were balanced based on their TN content and constants for each treatment in a given experimental series, including the control treatments. Nitrogen from PFM accounted for 50.6%, and the remaining 49.4% was introduced with urea (CO(NH_2_)_2_), except for series 1 (WBA), in which nitrogen was introduced only from CO(NH_2_)_2_. Constant NPK fertilization was used in the studies. Nitrogen rates from PFM were determined based on accepted agricultural practices limiting N introduced with organic fertilizers [5,6]. The total amounts of macronutrients used in the experiment were as follows: 0.112 g N, 0.067 g P and 0.134 g K kg^−1^ of soil, respectively. The doses of P and K were introduced into the soil in the form of aqueous solutions of potassium phosphate (KH_2_PO_4_) and potassium sulfate (K_2_SO_4_), respectively. All amendments (WBA, ULF, SSF, SLF and NPK) were incorporated into the soil according to the adopted scheme (Table 1). The soil with amendments was mixed and transferred to polyethylene (PE) pots. All experimental objects were carried out in 3 replicates.

Once the experiment was finished, the plant biomass was collected and a representative soil sample was taken from each pot. A total of 48 samples were obtained, with three replicates per experimental site (pot). The soil was air-dried, sieved through a 2 mm sieve, and then subjected to chemical analysis.

### 2.2. Characteristics of the Initial Soil and Materials (WBA, ULF, SSF and SLF)

The soil used in the experiment was taken from the humus layer of an arable field. Based on the granulometric composition (71.00% sand fraction (2.00–0.05 mm), 28.00% dust fraction (0.05–0.002 mm), and 1.00% clay fraction (<0.002 mm)), the soil was classified as loamy sand. The soil was very acidic (pH_KCl_ = 3.96), had low salinity (EC = 0.049 mS cm^−1^), and relatively low BS (61.60%) (Table 2). Among the available macronutrients in the soil, potassium (K_av_) dominated. WBA was waste obtained from the Municipal Heat Energy Company in Olsztyn, Poland. They are produced by burning exclusively wood chips, with a predominance of pine chips [10,37]. ULF, SSF, and SLF are by-products obtained from the Agricultural Biogas Plant in Łęguty, Poland. Due to the typical agricultural feedstock used in fermentation processes [37], all three PFM fractions constitute organic fertilizers in accordance with the adopted legal acts [5,7]. WBA, ULF, SSF, and SLF were characterized by an alkaline pH, in the range from 7.48 to 11.97 (Table 2). PFM was rich in TN (2.47–5.83 g kg^−1^) and TC (19.51–39.05 g kg^−1^), but the SSF had the richest composition in this range, also containing higher amounts of P_tot_ and Ca_tot_ than ULF and SLF. WBA contained similar amounts of TN (3.55 g kg^−1^) and Na_tot_ (2.18 g kg^−1^), but significantly higher amounts of TC (231.7 g kg^−1^), K_tot_ (33.41 g kg^−1^), Mg_tot_ (10.31 g kg^−1^) and Ca_tot_ (168.9 g kg^−1^) than PFM. The high Ca content in WBA resulted in its high alkalinity (26.93% CaO).

### 2.3. Description of Analytical Methods

In the materials used in the experiment, pH (pH_KCl_), TC, TN, total forms: P, K, Mg, Ca, and Na, HAC (hydrolytic acidity), and EC (electrolytic conductivity) were determined. In addition, total alkalinity was determined in the WBA.

In the soil, pH (pH_KCl_), TC, TN, available forms: P, K, and Mg, HAC, SBC (sum of base cations), and EC were determined. The CEC (cation exchange capacity) and BS (base saturation) were calculated from the obtained results, and the TC to TN ratio was calculated using the following formulas [38,39]:

CEC = SBC + HAC(1)

BS = (SBC/CEC) × 100%.(2)

Ratio = TC/TN(3)

The characteristics of the analytical methods used [40,41,42,43,44,45] and the equipment used are presented in Table S1.

### 2.4. Description of Statistical Methods

Statistical analysis included analysis of variance, Pearson’s correlation coefficient (r), and standard deviation (SD). The experiment was considered as a two-factor design, with increasing WBA doses as factor 1 and PFM fractions as factor 2. The LSD test was used to assess the influence of the factors under consideration using two-way ANOVA. Duncan’s test was used to determine homogeneous groups. In the tables and graphs, different letters indicate a significant effect (*p* ≤ 0.05, n = 3) of the studied factors. Lowercase letters (a–j) indicate the effect of the interaction between WBA doses and the PFM fraction on the analyzed element. Uppercase letters (A–D) indicate a significant effect of the WBA dose (considering the means for all series) or the PFM fraction (considering the means for all doses). The same letters placed next to numerical values indicate no significance for the considered factors. These calculations were performed using Statistica^®^ (version 13.3 PL; TIBCO Software Inc., Palo Alto, CA, USA) [46]. The direction of the effect of increasing WBA doses was assessed using Pearson’s coefficient, and statistical tables [47] allowed us to determine its significance. Coefficient values with *p* ≤ 0.05 were considered significant, and with *p* ≤ 0.01 as highly significant. SD and *r* calculations were performed using the program Microsoft Excel^®^ (version 2026; Microsoft, Redmond, WA, USA) [48].

## 3. Results

### 3.1. Reaction of Soil

**Figure 2:**
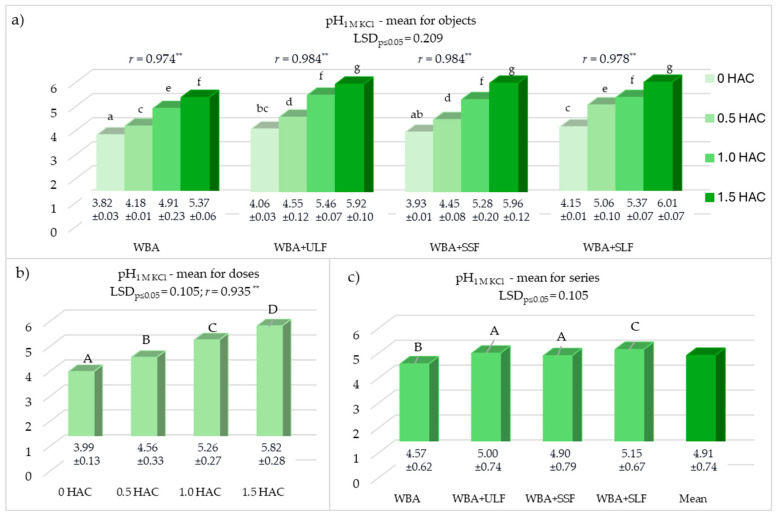
Soil reaction (pH_1MKCl_). HAC—hydrolytic acidity; WBA—woody biomass ash; ULF—unseparated liquid post-fermentation mass; SSF—separated solid post-fermentation mass; SLF—separated liquid post-fermentation mass; *r*—Pearson’s correlation coefficient; **—means significance at *p* ≤ 0.01; different letters indicate a significant effect (*p* ≤ 0.05, n = 3) of the studied factors: lowercase letters (a–g) the effect of interaction of WBA doses and PFM fraction on the pH value and uppercase letters (A–D) a significant effect of WBA dose or the used PFM fraction. (**a**) pH_1MKCl_—mean for objects; (**b**) pH_1MKCl_—mean for doses; (**c**) pH_1MKCl_—mean for series.

The average soil pH value after the experiment varied within a fairly wide range (3.82 ≤ pH_KCl_ ≤ 6.01) (Figure 2). The materials used in the study, both WBA and all PFM fractions (ULF, SSF, SLF), positively influenced the soil pH, significantly increasing the pH value (Figure 2a). The sole application of WBA caused a significant increase in pH, from 3.82 to 5.37 (*r* = 0.974 **), and the combined application of WBA and individual PFM fractions further intensified this effect (0.978 ** ≤ *r* ≤ 0.984 **) (Figure 2a). In the series in which both additives (WBA and PFM) were used, the pH value was significantly higher (4.90 ≤ pH_KCl_ ≤ 5.13) than in the objects with only WBA (pH_KCl_ = 4.57) (Figure 2c). The mean pH value for the doses (Figure 2b) indicated a highly positive effect of the combined use of WBA and PFM (*r* = 0.935 **). This effect was maintained regardless of the experimental series (Figure 2a) up to the highest applied dose of WBA (1.5 HAC). The most satisfactory results were obtained in series 4, in which WBA was used together with SLF (Figure 2c).

### 3.2. Content of TC, TN of Soil and Ratio TC/TN

The average TC content in the soil for all experimental series was 4.425 g kg^−1^ soil (Table 3). The obtained results prove a positive impact of the applied WBA doses on TC content (*r* = 0.474 **), with a significant effect noted for WBA doses 2 (1.0 HAC) and 3 (1.5 HAC). After application of these doses, compared to the control object, TC content in the soil increased by 5% and 4%, respectively. The combined application of WBA and PFM fractions also had a positive effect on TC content in the soil. However, the highest average TC content (4.533 g kg^−1^ soil) was recorded after the combined application of WBA and SSF (series 3) and was 5% higher compared to the series with WBA alone (series 1).

The factors considered did not significantly influence TN content in the soil (Table 3). The mean TN content in each experimental series was similar, ranging from 0.527 g in series 2 (WBA + ULF) to 0.555 g kg^−1^ of soil in series 4 (WBA + SLF). The WBA doses used, regardless of the experimental series, did not significantly affect TN content in the soil.

The observed increase in TC content in the soil after application of the adopted WBA doses also positively influenced the TC/TN ratio (Table 3). In series 1 (WBA), the ratio increased from 7.715 (control) to 8.792 (treatment with the highest WBA dose), as also indicated by the correlation coefficient (*r* = 0.720 **). Considering the average TC/TN ratio for all treatments, a significant effect was noted after the application of the second (1.0 HAC) and third doses of WBA (1.5 HAC). The second dose of WBA (1.0 HAC) had the most beneficial effect on the TC/TN ratio. Among the PFM fractions used, the combined application of WBA and ULF, as well as WBA and SSF, had a positive effect on the TC/TN ratio. However, only in the series 3 (WBA + SSF) had a significant effect, with the average TC/TN ratio being 8.465.

### 3.3. Soil Sorption Complex

**Figure 3:**
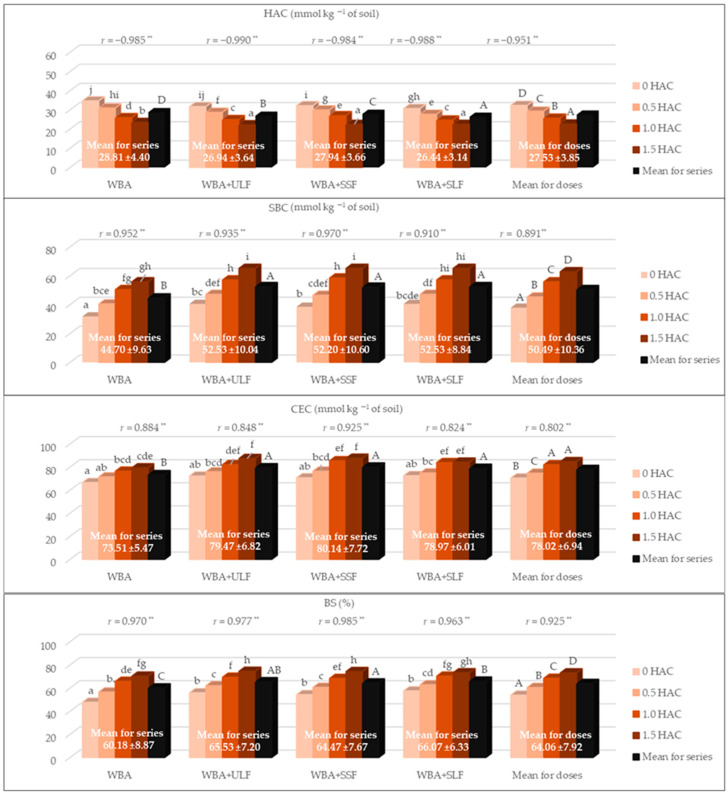
Soil sorption properties (HAC—hydrolytic acidity, SBC—sum of basic cations, CEC—cation exchange capacity and BS—base saturation). WBA—woody biomass ash; ULF—unseparated liquid post-fermentation mass; SSF—separated solid post-fermentation mass; SLF—separated liquid post-fermentation mass; *r*—Pearson’s correlation coefficient; **—means significance at *p* ≤ 0.01; different letters indicate a significant effect (*p* ≤ 0.05, n = 3) of the studied factors: lowercase letters (a–j) the effect of interaction of WBA doses and PFM fraction on the sorption complex properties and uppercase letters (A–D) a significant effect of WBA dose (mean for all series) or the used PFM fraction (mean for all doses).

The average HAC, SBC, and CEC values of the tested soil were 27.53, 50.49, and 78.02 mmol kg^−1^ of soil, respectively, and BS—64.04% (Figure 3). The combined application of WBA and PFM fractions significantly shaped the soil sorption complex. The adopted all WBA doses used significantly reduced HAC (*r* = −0.951 **), and increased SBC, CEC, and BS (0.802 ** ≤ *r* ≤ 0.925 **). After application of WBA alone to the soil (series 1), a 32% reduction in HAC was noted, while an increase in SBC and CEC was noted, respectively, by 75 and 19% compared to the control treatment. Consequently, BS increased from 47.80% to 70.37%. The combined application of WBA and PFM fractions resulted in similar changes. In these series, the HAC value decreased, while the remaining parameters of the sorption complex (SBC, CEC, BS) increased, as a result of increasing WBA doses. Considering the mean values of the sorption complex components in the individual experimental series, the combined use of WBA and PFM fractions had a more effective stabilizing effect on these characteristics. In these series, the mean HAC value was 3 to 8% lower than in series 1 (WBA). The mean SBC and CEC values were also more favorable in series 2 (WBA + ULF), 3 (WBA + SSF), and 4 (WBA + SLF), ranging from 17 to 18% (SBC) and 7 to 9% (CEC) higher than in series 1. In the series with the combined application of additives (WBA and PFM), the BS values were also more favorable. Taking into account the average BS value for individual series, the LSD test indicated WBA and SLF as the most optimal mixture, after the application of which the BS was 66.07%.

### 3.4. Content of Available Forms of Macronutrients (P_av_, K_av_ i Mg_av_)

**Figure 4:**
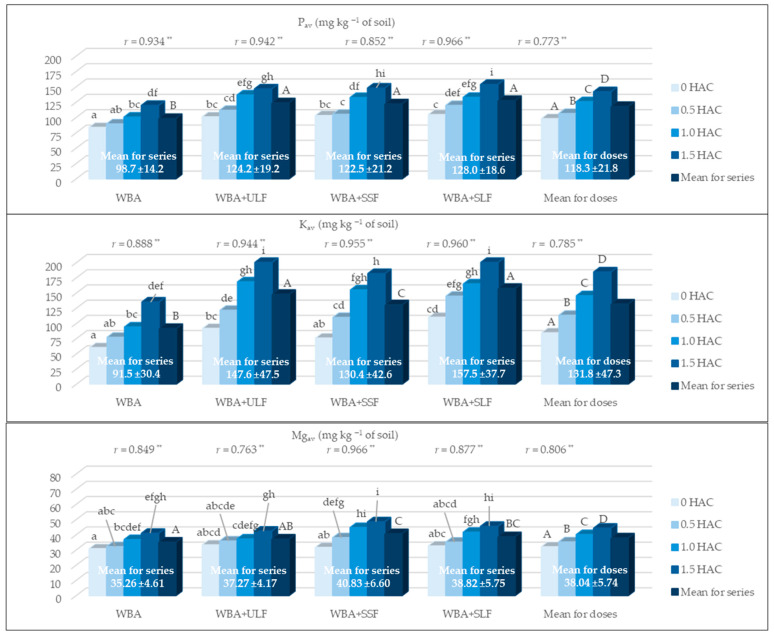
Content of available macronutrients (P_av_, K_av_, and Mg_av_) in the soil. HAC—hydrolytic acidity; WBA—woody biomass ash; ULF—unseparated liquid post-fermentation mass; SSF—separated solid post-fermentation mass; SLF—separated liquid post-fermentation mass; *r*—correlation coefficient; **—significant at *p* ≤ 0.01; different letters indicate a significant effect (*p* ≤ 0.05, n = 3) of the studied factors: lowercase letters (a–i) the effect of interaction of WBA doses and PFM fraction on the content of macronutrients and uppercase letters (A–D) a significant effect of WBA dose (mean for all series) or the used PFM fraction (mean for all doses).

The use of selected materials for the study, in addition to stabilizing pH and the sorption complex, also had a positive effect on the content of available macronutrients in the soil: Pav, Kav, and Mg_av_ (Figure 4). Among the available forms of these nutrients, the dominant content was K_av_ (131.8 mg) and P_av_ (118.3 mg kg^−1^ of soil). More than three times lower content was noted for Mg_av_, which was 38.04 mg Mg_av_ kg^−1^ of soil. The range of WBA doses used significantly increased the content of these nutrients (0.773 ** ≤ *r* ≤ 0.806 **). The highest increase in concentration, compared to increasing WBA doses, was observed for K_av_ (119%), while a significantly lower but still significant increase was observed for P_av_ (44%) and Mg_av_ (38%).

Combined application of WBA and PFM fractions enhanced the observed results. In the series in which PFM was added to WBA, the average P_av_ content was 24 to 30% higher than in the control series (WBA). Similarly, the average K_av_ content in the series with PFM was 42 to 72% higher, and Mg_av_ was 6 to 16% higher than in the series without these additives. The change in soil conditions in series 4 (WBA + SLF) had the most beneficial effect on P_av_ and K_av_ content, while in series 3 (WBA + SSF) on Mg_av_ content.

### 3.5. Soil Salinity

**Figure 5:**
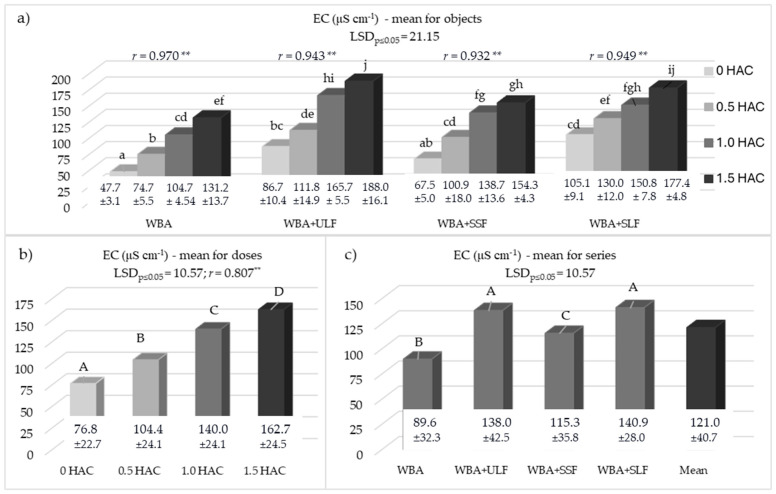
Electrolytic conductivity (EC) of soil: (**a**) mean for objects; (**b**) mean for doses; (**c**) mean for series. HAC—hydrolytic acidity; WBA—woody biomass ash; ULF—unseparated liquid post-fermentation mass; SSF—separated solid post-fermentation mass; SLF—separated liquid post-fermentation mass; *r*—Pearson’s correlation coefficient; **—means significance at *p* ≤ 0.01; different letters indicate a significant effect at *p* ≤ 0.05 (n = 3) of the studied factors: lowercase letters (a–j) the effect of interaction of WBA doses and PFM fraction on the EC value and uppercase letters (A–D) a significant effect of WBA dose or the used PFM fraction.

In addition to the positive effects of using WBA with PFM, an undesirable phenomenon was also observed—soil salinity, which was expressed by a significant increase in EC (Figure 5). An increase in soil salinity was observed in all series of the experiment across the entire range of WBA doses used (0.5–1.5 HAC) (0.932 ** ≤ *r* ≤ 0.970 **). The mean EC value for all experimental objects was 121.0 mS cm^−1^ (Figure 5c). By far the lowest mean EC value (86.6 mS cm^−1^) was found in control series (WBA), while in the series in which WBA and PFM were used, the EC value was significantly higher (Figure 5c). The highest EC value was found in the series in which, in addition to WBA, liquid PFM fractions (ULF and SLF) were used, and it was 138.0 and 140.9 mS cm^−1^, respectively.

### 3.6. Interrelationships Between the Described Features

Statistical analysis (Pearson’s coefficient) indicates that most of the discussed elements of the studied soil were highly significantly interrelated (Table 4). Among these close correlations, positive ones dominated (0.493 ** ≤ *r* ≤ 0.982 **), observed between components such as pH, EC, SBC, CEC, BS, TC, P_av_, K_av_, and Mg_av_. Highly significant, but negative relationships were noted between HAC and the remaining elements (−0.960 ** ≤ *r* ≤ −0.514 **). However, no significant relationships were noted between TN content and the other considered features.

## 4. Discussion

The factors considered in the current study, both the adopted WBA doses (0.5, 1.0, and 1.5 HAC) and the PFM fractions used (ULF, SSF, SLF), played a significant role in shaping basic soil properties, such as pH (Figure 2), sorption complex (Figure 3), and the content of available macronutrients (P_av_, K_av_, and Mg_av_) (Figure 4). As a result of using WBA alone, the soil pH value increased by 1.55 units (Figure 2). This effect was intensified by the application of PFM fractions to the soil, of which the SLF liquid fraction had the most positive effect. Regardless of the PFM fraction used, the increase in pH was observed up to the highest tested WBA dose (1.5 HAC) and amounted to 1.83 units compared to the control. The positive effect of WBA use on pH stabilization is also confirmed in the literature [10,49]. Similar relationships to those in the presented study were obtained after the use of WBA alone [10] and after the application of WBA with urea [49]. In turn, in the study, during the cultivation of perennial ryegrass and Indian mustard in slightly acidic soil, a dose of 10 t WBA ha^−1^ increased the soil pH by approximately 1 unit [17], similar to the effect obtained in our study in the object with second dose of WBA (1.0 HAC). The use of PFM alone also affects the increase in pH, which is observed in the control objects of presented studies and in the literature [11]. Therefore, the intensification of the pH improvement effect resulting from the combined use of WBA and PFM in the presented study resulted from the influence of both materials on this characteristic. In cucumber cultivation, the use of PFM and WBA [50] resulted in an increase in pH from 5.5 to even 7.5. These results correlate with our results, indicating the combined application of these materials as a more effective treatment in mitigating soil acidification. The increase in soil pH resulting from the use of the proposed treatments may have a positive effect on the absorption of nutrients [14]. However, the type of PFM may influence the acidity of the soil in various ways [51]. As indicated by the results of the presented studies and literature data [11], liquid forms are usually more effective.

The pH changes demonstrated in the present study clearly correlated strongly with changes occurring in the soil sorption complex. The increase in pH corresponded with a simultaneous decrease in HAC and an increase in parameters such as SBC, CEC, and BS, as evidenced by the correlation coefficient values (Table 4). A significant decrease in HAC and an increase in SBC was observed up to the highest WBA dose (1.5 HAC), which was additionally associated with an increase in BS (Figure 3). CEC increased after WBA application, but significantly only up to the second dose (1.0 HAC). In the series with the addition of PFM, the obtained results were more favorable. In the case of BS, the most favorable value was recorded in series 4 (WBA and SLF). In the study by Park et al. [28], no increase in CEC was observed at doses of 10 and 20 Mg WBA ha^−1^ of soil. This is a different result to that observed in our study. This difference may be due to the cultivation of different plant species, which absorb nutrients from the soil to varying degrees. The time of cultivation and the nature of the experiments were also certainly important. In our study, maize was tested for a short period in a pot experiment, while willow was tested for three years in the field experiment by Park et al. [28]. Some researchers [14] have pointed out that the physicochemical properties of soils after enrichment with WBA are comparable to those obtained using mineral fertilizers, and are sometimes even more favorable.

In our own studies, the increase in BS was probably due to the increased content of P_av_, K_av_, and Mg_av_ in the soil, which was associated not only with the applied WBA doses but also with the jointly applied PFM fractions (Figure 4). The results obtained indicate a synergistic effect of these two materials. Among the macronutrients considered, the content of K_av_ and P_av_ dominated, with the highest amounts in series 4 (WBA and SLF). However, the content of Mg_av_ was most beneficial with the joint application of WBA and SSF. This result is probably related to the fact that in all PFM fractions the content of K and P was significantly higher than that of Mg (Table 2). Mg availability is considered a key factor in controlling the amount of released N-NO_2_ [36]. The positive effect of WBA application demonstrated on the improvement of the soil sorption complex and the increase in the content of P_av_, K_av_, and Mg_av_ is also confirmed in the literature [10,49]. These authors observed similar results using WBA and urea as N supplementation. Przygocka-Cyna and Grzebisz [36] observed good N supply and a significant loss of K_av_ in the soil with increasing PFM doses when using PFM alone. Slightly different results were observed in studies by other authors [14] in which PFM and BA were used. After using these materials in field conditions, the authors noted an increase in the total content of P (by 15%), K (by 4%) and Mg (by 14%), as well as a decrease in the Ca content (by 4%). These different results are likely to be due to the different origins of the BA (from sorghum and Jerusalem artichoke biomass), the proportions of the two materials used and the nature of the experiment. These different observations indicate the need for further research in this area.

The WBA doses used in our study increased the TC content in the soil but had no effect on the TN content (Table 3). However, only the 2nd and 3rd doses of WBA (1.0 and 1.5 HAC) had a significant effect on the TC content and thus on the TC/TN ratio. Among the PFM fractions used, the combined application of WBA and SSF, as well as WBA and SLF, caused a significant increase in TC content, while the application of WBA and SSF contributed to an increase in TC/TN ratio. The increase in TC content in the current study, although significant, did not exceed 5% regardless of the factors. This may be due to the relatively low TC content in the materials used (Table 2), as also emphasized by other researchers [52]. PFM has a minor effect on the increase in TC content in the soil, which is related to soil depth and the type of fertilizer mixture [52]. It should be remembered that TC loss occurs during anaerobic fermentation [53]. The lack of increase in TN content is undoubtedly related to its small amount in the used WBA (Table 2), which is also confirmed by studies by other authors [17,28]. WBA typically contains low levels of N, as the majority of this component is released during the biomass combustion process [14]. In studies by other authors [14], an increase in total N content (by 20%) was observed after the use of PFM and BA. This result can be explained by the different origins of the ash, which affect its composition and its effect on the N content in the soil. Increasing the TN content in PFM can be achieved by adding legumes to silages used in fermentation processes [53]. In turn, the additional use of straw with PFM can increase the bio-immobilization of TN from PFM and simultaneously reduce the nitrate content in the soil [52]. The observed increase in the TC/TN ratio in our own studies is a very beneficial phenomenon, as it regulates many processes, for example, nitrogen release and its availability to plants and the decomposition of organic matter [53]. In series 3 (WBA + SSF), the average value of the TC/TN ratio was 8.465 (Table 3) and the optimal value on arable land is 10–13:1 [53]. Therefore, in further studies, the addition of substances containing higher amounts of TC should be considered.

In summarizing these positive results, it is also necessary to address the negative effects, which undoubtedly included a significant increase in EC (Figure 5). This increase in this parameter was observed with each of the WBA doses, which was intensified by the interaction of WBA with PFM fractions, of which the liquid fractions (ULF and SLF) had the most intense effect. This increase in EC indicates that both WBA and PFM may contribute to soil salinization, which may pose a risk in long-term use. The liquid fractions (ULF and SLF) had a greater effect due to the content of components in forms less bound to PFM components, which enriched the sorption complex more quickly. Previous studies [11], which used only PFM, obtained different results, as solid forms of PFM had a more intense effect on soil salinity than liquid forms. This may be due to the length of the plant’s persistence period as well as the origin of the PFM. In the present study, although salinity increased, EC values were very low and did not pose a threat to cultivated plants. Similar observations were made by other researchers [54] who, using PFM, noted an increase in soil EC, but these values remained acceptable for plant growth. Slightly different results were observed in studies using PFM and biochar, applied to the soil at 5% and 1% [31]. In these studies, a significant increase in soil salinity was noted after PFM application, while the combined application of PFM and biochar mitigated this process. This suggests that the selection of components for the fertilizer mixture is important. The increase in salinity observed in previous studies considering a wider range of individually applied WBA and PFM doses [10,11] indicates that further increases in the doses of these materials used together may result in increased soil salinity. It should also be expected that maintaining the currently proposed PFM doses will not mitigate the effects of soil salinization resulting from the use of increased WBA doses.

In agricultural use of WBA, the method of application is important. It is generally recommended to apply WBA before sowing, and it should be spread and evenly mixed with the topsoil. The most beneficial WBA dose is 1 to 5 t ha^−1^. At higher doses, the content of P_av_ and K_av_ in the soil would be too high, which could result in leaching from the topsoil due to the lack of necessary colloid content. In nitrogen-poor soils, it is recommended to combine WBA with the addition of nitrogen-rich substances [17]. In the presented studies, although WBA and PFM were used together, they were added to the soil as separate materials, which helped to avoid nitrogen reduction to the ammonium form and carbon dissolution [55,56]. Properly preparing the WBA/PFM mixture offers the opportunity to prepare a slow-release fertilizer. To ensure optimal results from the combined use of WBA and PFM, the doses and application methods of these additives must be precisely selected [31], as well as the WBA to PFM ratio [51]. When using such a mixture in poplar fertilization, the most appropriate ratio is 1–2:1 (one- or two-parts WBA and one part PFM) [51]. However, in cucumber cultivation [50], the optimal WBA to PFM ratio was 1:4. According to some researchers [51], the origin of WBA and the type of PFM do not affect the effectiveness of the mixture. In their opinion, only a sufficient nutrient content is important, as long as both substrates come from reliable sources. The usefulness of the WBA and PFM mixture for fertilization purposes is also confirmed by the results obtained by Adamovičs and Poiša [57]. These authors concluded that using these materials together can improve soil fertility and achieve high and good-quality winter wheat yields without the use of mineral fertilizers. Furthermore, drying PFM may be unprofitable, and adding WBA during PFM dewatering allows for soil liming and enrichment with nutrients [50]. To improve nutrient immobilization, some consider processes such as carbonation, calcination, acidification, washing, grinding, and sieving. They suggest a 5:1 ratio of WBA to PFM, which allows to produce a stable granular fertilizer with the best mechanical properties [56]. Developing a slow-release fertilizer mixture (with WBA and PFM) would be consistent with the principles of a circular economy. To make this feasible and safe, a method for reducing nitrogen loss during the mixing process and at the end of incubation must also be developed. The high pH of both materials favors the conversion of organic nitrogen to ammonium but simultaneously reduces CO_2_ release. To avoid N losses, H_2_SO_4_ traps can be used [55]. Mineralization of organic nitrogen in the soil because of WBA use may increase N uptake by plants [20].

On the one hand, WBA is a valuable fertilizer ingredient because it contains significant amounts of nutrients, e.g., Ca, K and microelements. On the other hand, however, it may contain toxic compounds harmful to the environment, including heavy metals [19,20,27], especially Cd [18,20] and Cr [27], as well as substances resulting from combustion (PHAs—polycyclic aromatic hydrocarbons or VOCs—volatile organic compounds). Furthermore, we can expect the presence of chlorides and sulfates in WBA composition. If WBA is characterized by low levels of contamination, it should be used in agriculture and for the reclamation of degraded land. As a result of WBA deposition in landfills, valuable ingredients are lost [3]. Therefore, before deciding on the environmental management of this waste, a series of analyses and studies should be performed to assess not only the composition of WBA but also its impact on the soil and potential yields. The dynamics of elements in the soil-solution-crop systems after WBA application should be thoroughly assessed. To assess the cumulative impact of WBA on soil, as well as crop quality and environmental sustainability, long-term field studies are necessary [27]. It should be noted that the number of heavy metals introduced into the soil from WBA can be increased by the joint use of this material with PFM, which is also a source of these elements [11,36].

Nutrient recycling through the soil application of PFM and WBA helps close the nutrient cycle and simultaneously solves the problem of their improper management [33] and reduces the costs of storing these materials [17]. Furthermore, WBA can be used to condition sewage sludge by significantly dewatering it and reducing the number of bacteria. This results in obtaining valuable fertilizer while reducing the costs of sludge dewatering. This fertilizer is particularly recommended for perennial plantations [58]. To fully utilize the benefits of WBA, it is necessary to carefully evaluate its content of nutrients, not only those for fertilizer purposes but also those for other applications, such as cementitious materials and geopolymers [4].

The research conducted does not clearly indicate that one PFM fraction is most effective in improving basic soil properties. However, due to the identified problem of post-process quenching of WBA and the additional costs associated with drying PFM, a reasonable solution would be to use the initial form (ULF). Selecting this fraction would be an economically viable solution for both plants, due to the savings generated by not drying PFM and quenching WBA. Furthermore, with systematic research into soil salinity and supplementing it with TN, this solution could be safe for the environment and potential crops and would allow for closing the cycle of elements contained in biomass intended for energy purposes, as well as in biodegradable waste for biogas purposes. Alternatively, biogas plants could consider drying the initial PFM and then using the liquid fraction (SLF) to create a mixture with WBA and distributing the dried fraction (SSF) as a separate type of organic fertilizer.

Given the positive impact observed in the combined use of WBA and PFM on basic soil properties, it is important to note the increase in electrolytic conductivity observed in the present study, which results in soil salinization. In the long term of combined use of both materials, emphasis should be placed on systematically examining not only the composition of these materials but also of the soil on which they are to be applied.

## 5. Conclusions

The factors considered in the experiment proved crucial in shaping basic soil properties, which must be maintained at appropriate levels when maintaining soil health or initiating stabilization processes. Both the application of WBA alone and the combined use of WBA with the tested PFM fractions (ULF, SSF, SLF) resulted in significant increased pH, SBC, CEC, and BS values, while simultaneously reducing the HAC value, which is significant in acidic soils. The addition of WBA and the PFM fraction significantly increased the soil content of TC (by 4%), P_av_ (by 44%), K_av_ (by 119%), and Mg_av_ (by 38%), elements important for plant nutrition, which may be crucial in processes related to planned remediation treatments involving plants. The negative side of WBA use is the negligible amount of TN and increased soil salinity (on average about 112%), which is exacerbated by the addition of PFM, particularly the liquid fractions (ULF, SLF). When considering the use of the three adopted treatments on a larger scale, it is important to remember to test the soil for salinity before carrying out the treatment and to adjust the doses of both materials (WBA, PFM) so as not to exceed safe thresholds and to secure the supply of TN. It should be remembered that increasing the doses used in the current experiment may result not only in enriching the sorption complex with alkaline cations but also in a further increase in soil salinity.

## Figures and Tables

**Table 1:** Experimental design.

Series of Experiment			
WBA (Doses of HAC)	WBA + ULF	WBA + SSF	WBA + SLF
0 WBA + NPK	0 WBA + ULF + NPK	0 WBA + SSF + NPK	0 WBA + SLF + NPK
0.5 WBA + NPK	0.5 WBA + ULF + NPK	0.5 WBA + SSF + NPK	0.5 WBA + SLF + NPK
1.0 WBA + NPK	1.0 WBA + ULF + NPK	1.0 WBA + SSF + NPK	1.0 WBA + SLF + NPK
1.5 WBA + NPK	1.5 WBA + ULF + NPK	1.5 WBA + SSF + NPK	1.5 WBA + SLF + NPK

WBA—woody biomass ash; ULF—unseparated post-fermentation mass; SSF—separated post-fermentation mass; SLF—unseparated post-fermentation mass; HAC—hydrolytic acidity; NPK—mineral fertilizers added in the form of liquid urea solutions (CO(NH_2_)_2_), potassium sulfate (K_2_SO_2_) and potassium phosphate (KH_2_PO_2_).

**Table 2:** Selected properties of the starting soil and materials (WBA, ULF, SSF and SLF).

Parameter	Unit	Starting Soil	WBA	ULF	SSF	SLF
Dry mas	%	-	60.28 ± 0.65	5.51 ± 0.24	24.56 ± 0.62	5.24 ± 0.04
Soil reaction (pH_KCl_)	−log_10_(H^+^)	3.96 ± 0.02	11.97 ± 0.09	7.48 ± 0.03	9.53 ± 0.03	7.49 ± 0.03
Electrical conductivity (EC)	mS cm^−1^	0.05 ± 0.00	10.49 ± 0.23	29.13 ± 1.04	25.15 ± 1.12	28.87 ± 0.94
Hydrolytic acidity (HAC)	mmol kg^−1^	28.25 ± 1.02	600.0 ± 4.08	285.0 ± 8.50	116.3 ± 3.75	300.0 ± 5.00
Sum of base cations (SBC)	mmol kg^−1^	45.33 ± 0.61	-	-	-	-
Cation exchange capacity (CEC)	mmol kg^−1^	73.58 ± 1.63	-	-	-	-
Base saturation (BS)	%	61.60 ± 0.53	-	-	-	-
Total carbon (TC)	g kg^−1^	4.09 ± 0.09	231.7 ± 8.33	20.95 ± 0.12	39.05 ± 1.34	19.51 ± 0.58
Total nitrogen (TN)	g kg^−1^	0.54 ± 0.01	3.55 ± 0.33	2.80 ± 0.11	5.83 ± 0.18	2.47 ± 0.18
Total macronutrients:						
Phosphorus (P_tot_)	g kg^−1^	-	10.51 ± 0.33	2.11 ± 0.16	13.36 ± 0.49	2.05 ± 0.07
Potassium (K_tot_)	g kg^−1^	-	33.41 ± 0.13	8.56 ± 0.06	7.72 ± 0.21	8.36 ± 0.13
Magnesium (Mg_tot_)	g kg^−1^	-	10.31 ± 0.05	0.38 ± 0.00	0.48 ± 0.00	0.37 ± 0.01
Calcium (Ca_tot_)	g kg^−1^	-	168.9 ± 0.51	2.37 ± 0,10	4.77 ± 0.02	2.14 ± 0.23
Sodium (Na_tot_)	g kg^−1^	-	2.18 ± 0.02	3.41 ± 0.07	3.25 ± 0.10	3.51 ± 0.12
Available macronutrients:						
Phosphorus (P_av_)	mg kg^−1^	68.63 ± 4.38	-	-	-	-
Potassium (K_av_)	mg kg^−1^	97.69 ± 1.70	-	-	-	-
Magnesium (Mg_av_)	mg kg^−1^	31.00 ± 1.13	-	-	-	-
Alkalinity	% CaO	-	26.93 ± 0.88	-	-	-

WBA—woody biomass ash; ULF—unseparated post-fermentation mass; SSF—separated post-fermentation mass; SLF—unseparated post-fermentation mas.

**Table 3:** Content of TC (total carbon) and TN (total nitrogen) and the TC/TN ratio value.

WBA Doses of HAC	Series of Experiment				
	WBA	WBA + ULF	WBA + SSF	WBA + SLF	Mean for Doses
TC (g kg^−1^ of soil)					
0	4.169 ± 0.096 a	4.296 ± 0.088 ab	4.393 ± 0.087 abcd	4.308 ± 0.067 abc	4.292 ± 0.117 A
1	4.198 ± 0.090 a	4.328 ± 0.147 abc	4.483 ± 0.103 bcde	4.457 ± 0.047 bcde	4.367 ± 0.154 AB
2	4.503 ± 0.127 bcde	4.475 ± 0.118 bcde	4.693 ± 0.070 e	4.608 ± 0.187 de	4.570 ± 0.158 C
3	4.378 ± 0.070 abcd	4.545 ± 0.142 bcde	4.563 ± 0.125 cde	4.409 ± 0.074 abcd	4.474 ± 0.135 BC
Mean for series	4.312 ± 0.168 A	4.411 ± 0.162 AB	4.533 ± 0.148 C	4.445 ± 0.153 BC	4.425 ± 0.177
*r*	0.621 ^n.s.^	0.616 ^n.s.^	0.546 ^n.s.^	0.330 ^n.s.^	0.474 **
TN (g kg^−1^ of soil)					
0	0.541 ± 0.017 abc	0.527 ± 0.026 abc	0.532 ± 0.023 abc	0.579 ± 0.013 c	0.545 ± 0.029 A
1	0.551 ± 0.024 abc	0.546 ± 0.000 abc	0.518 ± 0.030 ab	0.523 ± 0.026 abc	0.534 ± 0.027 A
2	0.551 ± 0.013 abc	0.513 ± 0.037 ab	0.551 ± 0.013 abc	0.551 ± 0.053 abc	0.541 ± 0.037 A
3	0.499 ± 0.024 a	0.523 ± 0.017 abc	0.546 ± 0.023 abc	0.569 ± 0.013 bc	0.534 ± 0.033 A
Mean for series	0.536 ± 0.029 AB	0.527 ± 0.027 A	0.537 ± 0.026 AB	0.555 ± 0.038 B	0.539 ± 0.032
*r*	−0.482 ^n.s.^	−0.193 ^n.s.^	0.316 ^n.s.^	−0.017 ^n.s.^	−0.085 ^n.s.^
TC/TN (ratio)					
0	7.715 ± 0.425 abc	8.162 ± 0.336 abcd	8.273 ± 0.418 abcd	7.448 ± 0.160 a	7.900 ± 0.485 B
1	7.631 ± 0.189 ab	7.926 ± 0.270 abcd	8.683 ± 0.524 d	8.548 ± 0.433 cd	8.197 ± 0.575 AB
2	8.178 ± 0.187 abcd	8.752 ± 0.501 d	8.526 ± 0.166 bcd	8.416 ± 0.505 bcd	8.468 ± 0.430 A
3	8.792 ± 0.523 d	8.704 ± 0.384 d	8.377 ± 0.492 bcd	7.746 ± 0.126 abc	8.405 ± 0.582 A
Mean for series	8.079 ± 0.586 AB	8.386 ± 0.520 AB	8.465 ± 0.451 B	8.039 ± 0.575 A	8.242 ± 0.567
*r*	0.720 **	0.527 ^n.s.^	0.038 ^n.s.^	0.148 ^n.s.^	0.352 *

HAC—hydrolytic acidity; WBA—woody biomass ash; ULF—unseparated liquid post-fermentation mass; SSF—separated solid post-fermentation mass; SLF—separated liquid post-fermentation mass; TC—total carbon; TN—total nitrogen; *r*—correlation coefficient; **—significant at *p* ≤ 0.01; *—significant at *p* ≤ 0.05; ^n.s.^—not significant; different letters indicate a significant effect (*p* ≤ 0.05, n = 3) of the studied factors: lowercase letters (a–e) the effect of interaction of WBA doses and PFM fraction on the content of TC, TN and ratio TC/TN and uppercase letters (A–C) a significant effect of WBA dose (mean for all series) or the used PFM fraction (mean for all doses).

**Table 4:** Correlation coefficient (*r*) between elements.

Elements	pH	EC	TC	TN	P_av_	K_av_	Mg_av_	HAC	SBC	CEC
EC	0.922 **									
TC	0.523 **	0.493 **								
TN	−0.030	−0.010	0.134							
P_av_	0.899 **	0.893 **	0.493 **	0.053						
K_av_	0.921 **	0.954 **	0.460 **	0.011	0.942 **					
Mg_av_	0.820 **	0.731 **	0.452 **	−0.026	0.803 **	0.764 **				
HAC	−0.960 **	−0.885 **	−0.514 **	0.069	−0.841 **	−0.856 **	−0.787 **			
SBC	0.935 **	0.897 **	0.558 **	−0.051	0.864 **	0.889 **	0.846 **	−0.928 **		
CEC	0.863 **	0.848 **	0.547 **	−0.038	0.823 **	0.853 **	0.826 **	−0.830 **	0.978 **	
BS	0.954 **	0.908 **	0.559 **	−0.060	0.858 **	0.883 **	0.826 **	−0.974 **	0.982 **	0.926 **

EC—electrolytic conductivity; TC—total carbon; TN—total nitrogen; HAC—hydrolytic acidity; SBC—sum of basic cations; CEC—cation exchange capacity; BS—base saturation; **—Pearson’s correlation coefficient (r) significant at *p* ≤ 0.01; 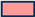
—positive correlations; 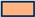
—negative correlations.

## Data Availability

The original contributions presented in this study are included in the article/Supplementary Materials. Further inquiries can be directed to the corresponding author.

## Supplementary Materials

The following supporting information can be downloaded at: https://www.mdpi.com/article/10.3390/ma18225176/s1, Table S1: Characteristics of the methods and apparatus used.

## Abbreviations

The following abbreviations are used in this manuscript:

WBA	woody biomass ash
PFM	post-fermentation mass
ULF	unseparated liquid post-fermentation mass
SSF	separated solid post-fermentation mass
SLF	separated liquid post-fermentation mass
