# Supplementary material for: Wood Biomass Ash (WBA) Used in Conjunction with Post-Fermentation Mass (PFM) as a Way to Stabilize Soil Properties
Source: Materials (Basel). 2025 Nov 14;18(22):5176. doi: 10.3390/ma18225176 (PMC12654451; doi:10.3390/ma18225176)
Supplement: Supplementary file 1 [file materials-18-05176-s001.zip › materials-3942961-supplementary.pdf]

materials

Article

Wood biomass ash (WBA) used in conjunction with post-fermentation mass (PFM) as a way to stabilize soil properties

Elżbieta Rolka *, Mirosław Wyszkowski, Andrzej Cezary Żołnowski, Anna Skorwider-Namiotko and Radosław Szostek

Supplementary materials

Table S1. Characteristics of the methods and apparatus used

Elements	Materials	Methods, Apparatus
Soil Particle Size Distribution	Soil	Laser method; Mastersizer 3000, Hydro EV module (Malvern Instruments, Worcestershire, UK) [40].
Reaction (pH_{KCl})	Soil WBA, ULF, SSF, SLF	Potentiometric method; 1M KCl; pH 538 laboratory pH meter, WTW electrode (WTW, Wrocław, Poland) [41].
Electric conductivity (EC)	Soil, WBA, ULF, SSF, SLF	Conductivity method; HANNA HI8733 conductivity meter (Hanna Instruments, Leighton Buzzard, United Kingdom) [41].
Sum of base cations (SBC)	Soil	Kappen method [41].
Hydrolytic acidity (HAC)	Soil, WBA, ULF, SSF, SLF	Kappen method [41].
Total carbon (TC)	Soil, WBA, ULF, SSF, SLF	TOC Analyser (Shimadzu Corporation, Kyoto, Japan), SSM-5000A adapter (Solid Sample Module, Shimadzu Corporation, Kyoto, Japan).
Total nitrogen (TN)	Soil, WBA, ULF, SSF, SLF	Kjeldahl distillation method [42]; concentrated sulfuric acid (VI) with hydrogen peroxide; wet digestion - Speed Digester K-439 digestion furnace (BÜCHI Labor-technik AG, Flawil, Switzerland), scrubber K-415 vapor absorber (BÜCHI Labor-technik AG, Flawil, Switzerland), distillation - K-355 steam still (BÜCHI Labor-technik AG, Flawil, Switzerland).
Total forms: P_{tot}, K_{tot}, Mg_{tot}, Ca_{tot}, and Na_{tot}	WBA, ULF, SSF, SLF	P_{tot} - vanadium-molybdenum method [43]. Mg_{tot} - atomic absorption spectrometry (AAS) and K_{tot}, Ca_{tot}, Na_{tot} - flame atomic emission spectroscopy (FAES) [43]; AA240FS Fast Sequential Atomic Absorption Spectrometer (Varian Inc., Mulgrave, Australia). These elements were determined on the same mineralized samples used for the $N_{tot.}$
Available forms: P_{av}, K_{av} and Mg_{av}	Soil	P_{av} and K_{av} - Egner-Riehm method [44]. Mg_{av} - Schachtschabel method [43].
Alkalinity (% CaO)	WBA	Method used to assess the alkalinity of calcium and calcium-magnesium fertilizers [45].
